# Isocenter Optimization in WBRT: Concurrent Sparing of Lens and Lacrimal Gland via Anterior Penumbra Sharpening

**DOI:** 10.1002/acm2.70535

**Published:** 2026-03-18

**Authors:** Chen Xie, Xuemei Xia, Haowen Pang, Yan Zhang, Lin Li, Simin Lu

**Affiliations:** ^1^ Department of Oncology Luzhou People's Hospital Luzhou Sichuan China; ^2^ Department of Plastic and Burn Surgery The Affiliated Hospital of Southwest Medical University Luzhou Sichuan China; ^3^ Department of Oncology The Affiliated Hospital of Southwest Medical University Luzhou Sichuan China

**Keywords:** field‐in‐field technique (FIF), isocenter optimization, lacrimal gland, lens, three‐dimensional conformal radiotherapy (3D‐CRT), whole‐brain radiotherapy (WBRT)

## Abstract

**Background:**

With improving survival for patients receiving whole‐brain radiotherapy (WBRT), mitigating long‐term toxicities like cataract and dry eye syndrome has become increasingly critical. The lens and lacrimal gland are highly radiosensitive and lie in close proximity to the target volume, posing a persistent challenge for achieving sharp dose gradients. While modern techniques like multileaf collimator (MLC) shaping offer some protection, the potential of classic geometric optimization principles, such as anterior beam shift, remains underexplored in contemporary treatment planning workflows.

**Purpose:**

This study aimed to systematically translate, quantitatively validate, and integrate the classic geometric principle of anterior isocenter shift into the modern treatment planning workflow for the dual protection of the lens and lacrimal gland in WBRT, utilizing 3D‐conformal radiotherapy (3D‐CRT) and field‐in‐field (FIF) techniques.

**Methods:**

For 40 patients, conventional and isocenter‐optimized plans (involving an anterior shift of the isocenter within the PTV) were generated for both 3D‐CRT and FIF. We compared dosimetric parameters for the planning target volume (PTV), lenses, lacrimal glands, and other organs at risk. Plan quality, normal tissue complication probability (NTCP) for cataract and dry eye syndrome, and clinical risk stratification were evaluated

**Results:**

Isocenter optimization significantly reduced the median lens Dmax by 20% and PRV_lens D0.03 cm3 by 23% (*p *< 0.001). Lacrimal gland Dmean, V6Gy, and V10Gy were also significantly reduced. The strategy physically improved the dose gradient, narrowing the penumbra width by 31.25% and increasing the dose fall‐off rate by 15%, while reducing low‐dose irradiation volumes outside the PTV. These dosimetric benefits translated into meaningful reductions in projected NTCP for both complications. The 3D‐FIF plans maintained target coverage and homogeneity, mitigating the heterogeneity increase observed in 3D‐CRT plans.

**Conclusion:**

Anterior isocenter optimization is a practical, and hardware‐free technique that seamlessly synergizes with modern MLC‐based planning to provide significant, concurrent sparing of the lens and lacrimal gland in WBRT. As a readily implementable modification within existing planning systems, this strategy can be adopted immediately to enhance treatment safety without requiring additional resources.

## 1 INTRODUCTION

Whole‐brain radiotherapy (WBRT) remains a cornerstone treatment for brain metastases^1^, ^2^ and other specific central nervous system malignancies,^3^, ^4^, ^5^, ^6^ including prophylactic cranial irradiation for small cell lung cancer.^7^, ^8^ With patient survival now extending from years to decades across these indications,^9^, ^10^, ^11^ managing long‐term toxicities has become a critical determinant of quality of life.

Among the various delivery techniques, three‐dimensional conformal radiotherapy (3D‐CRT) and field‐in‐field (FIF) are widely adopted due to their operational efficiency, cost‐effectiveness, and clinically acceptable dose distributions.^12^, ^13^, ^14^, ^15^, ^16^ Critical organs at risk (OARs) include the lenses,^17^, ^18^ parotid glands,^19^, ^20^ lacrimal glands,^21^, ^22^ and skin,^23^, ^24^ alongside concerns for secondary malignancies.^18^

The lens is exceptionally radiosensitive,^25^ with even low doses of ionizing radiation capable of initiating a pathogenic cascade leading to posterior subcapsular cataract. A well‐established dose‐response relationship exists, linking elevated lens dose to increased cataract incidence and shorter latency (typically 2–8 years).^26^, ^27^, ^28^ Consequently, the International Commission on Radiological Protection (ICRP) recommends a stringent dose constraint of Dmax < 5 Gy,^29^ though emerging data suggest risks may persist even below 2 Gy,^30^, ^31^ reinforcing the need to minimize exposure.

Similarly, the lacrimal gland is highly vulnerable. Its proximity to the target volume results in substantial irradiation during conventional WBRT, contributing to a significant incidence of acute, dose‐volume‐dependent dry eye syndrome.^21^, ^22^, ^32^ While prospective delineation and technical modifications like MLC shielding can markedly reduce dose,^21^ dedicated studies exploring optimized sparing strategies remain limited.

The anatomical proximity of these ocular structures (within 4–8 mm of the PTV) presents a fundamental and persistent challenge for achieving sharp dose gradients.^33^, ^34^ Notably, this challenge was addressed in the 2D radiotherapy era through two distinct principles: physical penumbra trimming (e.g., close‐proximity hanging blocks^34^) and geometric optimization (e.g., anterior shift of the beam's central axis^35^). The underlying mechanism of the latter is the sharpening of the anterior transmission penumbra: by reducing the beam's angle of incidence relative to the multileaf collimator (MLC) leaf ends, the dose gradient is mechanically compressed, with the physical optimum achieved when the central axis (or, in 3D planning, the isocenter) is positioned at the anterior target edge. The transition to 3D‐conformal radiotherapy seamlessly incorporated the first principle into modern practice as MLC shaping with jaw blocking. However, the second principle—geometric optimization through beam shifting—has not been systematically quantified or operationalized within the contemporary digital treatment planning workflow. This study aims to bridge this gap by providing a quantified, ready‐to‐implement protocol. Consequently, the potential incremental benefit of integrating this geometric principle with modern MLC‐based techniques remains unquantified, particularly for concurrent sparing of adjacent ocular structures.

Therefore, a strategy that concurrently mitigates these toxicities is highly desirable. To address this opportunity for optimization and translate the principle into a clinical tool, this study aims to develop and quantitatively evaluate a ready‐to‐implement protocol for anterior isocenter shift to achieve concurrent sparing of the lens and lacrimal gland in modern WBRT utilizing 3D‐CRT and FIF techniques (See Tables 1 and 2).

**TABLE 1:** Comparison of conventional versus optimized 3D‐CRT plans (selected parameters).

Parameter	Unit	3D‐Conv	3D‐New	difference(95%CI)	*t/Z*	*P*‐value	Effect size
**Target**							
**PTV_V_28.5Gy_**	%	99.8[99.8,99.9]	99.7[99.6,99.7]	0.1(0.08→0.22)	5.531	<0.001^**^	*r = 0.9*
**PTV_V_33Gy_**	%	0.9[0.3,1.5]	7.9[4,14.7]	−7(−15.91→−0.79)	−5.484	<0.001^**^	*r* = −0.87
**PTV_D_2%_**	Gy	32.8[32.6,32.9]	33.5[33.2,33.7]	−0.7(−0.85→−0.55)	−5.539	<0.001^**^	*r *= −0.88
**PTV_D_max_**	Gy	34.2[33.9,34.3]	34.8[34.6,35]	−0.9(−1→−0.4)	−5.5	<0.001^**^	*r *= −0.87
**OARs**							
**Lens_D_max_**	Gy	4.5[3.9,5.225]	3.6[3.4,4]	0.9(0.45→1.35)	5.521	<0.001^**^	*r *= 0.87
**Lens_D_mean_**	Gy	3.1[2.9,3.275]	2.6[2.5,2.7]	0.5(0.48→0.62)	5.604	<0.001^**^	*r *= 0.89
**P Lens_D_mean_**	Gy	3.3[3.1,3.6]	2.7[2.6,3]	0.6(0.45→0.75)	5.557	<0.001^**^	*r *= 0.88
**P Lens_D_max_**	Gy	9.9 ± 2.7	8.3 ± 2.9	1.6(1.31→1.91)	10.875	<0.001^**^	*d *= 1.78
**P Lens_D_0.03_**	Gy	7.4 ± 2.1	5.7 ± 1.9	1.7(1.49→1.92)	15.812	<0.001^**^	*d *= 2.43
**Lacrimal_D_mean_**	Gy	8.4 ± 1.9	7.5 ± 2	0.9(0.79→1.07)	13.115	<0.001^**^	*d *= 2.25
**Lacrimal_V_6Gy_**	%	55.7 ± 13.7	37.1 ± 11.9	18.6(17.01→20.1)	24.305	<0.001^**^	*d *= 3.88
**Lacrimal_V_10Gy_**	%	32.6 ± 12.1	23.7 ± 10.3	8.9(7.64→10.05)	14.851	<0.001^**^	*d *= 2.35
**Lacrimal_V_15Gy_**	%	11.5 ± 8.6	14.4 ± 8.4	−2.9(−3.49→−2.2)	−8.925	<0.001^**^	*d *= −1.4
**Plan Quality**							
**Dr**	Gy/mm	2[1.9,2.2]	2.3[2.,2.5]	−0.3(−0.45→−0.05)	−5.26	<0.001^**^	*r *= −0.88
**Pb_Eye**	mm	8[8,8.5]	5.5[5.5,6]	2.5(1.84→3.16)	5.559	<0.001^**^	*r *= 0.88
**HI**		0.094 ± 0.007	0.117 ± 0.009	−0.023(−0.024→−0.021)	−30.102	<0.001^**^	*d *= −4.6
**V_5Gy‐PTV_**	cc	1237.9 ± 163.1	1196.4 ± 157.1	41.5(38.86→44.13)	31.827	<0.001^**^	*d *= 5.03
**V_2Gy‐PTV_**	cc	1666.8 ± 219.7	1542.4 ± 206.7	124.4(119.12→129.54)	48.282	<0.001^**^	*d *= 7.63

***Notes***: Data: Mean ± SD (normal) or Median [IQR] (non‐normal).

Full parameters: Supplementary Tables S3.

** *P *< 0.00156 (Bonferroni‐corrected).

**TABLE 2:** Comparison of conventional versus optimized 3D‐FIF plans (selected parameters).

Parameter	Unit	FIF‐Conv	FIF‐New	difference(95%CI)	*t/Z*	*P‐*value	Effect size
**Target**							
**PTV_V_30Gy_**	%	96.9[96.5,97]	96.8[96.5,97]	0.1(−0.15→0.15)	0.703	0.482	*r *= 0.12
**PTV_V_28.5Gy_**	%	99.8[99.7,99.9]	99.7[99.6,99.8]	0.1(−0.05→0.25)	4.796	<0.001^**^	*r *= 0.89
**PTV_V_33Gy_**	%	0[0,0]	0[0,0]	0(0→0)	0	1	0
**PTV_D_2%_**	Gy	32.2[32,32.3]	32.2[32.1,32.3]	0(−0.22→0.04)	−3.002	0.003	*r *= −0.57
**PTV_D_max_**	Gy	33.2[33,33.3]	33.2[33,33.3]	0(−0.15→0.15)	−0.044	0.965	*r *= −0.01
**OARs**							
**Lens_D_max_**	Gy	4.5[4.1,5.4]	3.6[3.4,4.2]	0.9(0.45→1.35)	5.47	<0.001^**^	*r *= 0.86
**Lens_D_mean_**	Gy	3.1[3,3.4]	2.7[2.5,2.9]	0.4(0.35→0.65)	5.569	<0.00^1**^	*r *= 0.88
**P Lens_D_mean_**	Gy	3.4[3.2,3.7]	2.8[2.7,3]	0.6(0.45→0.75)	5.56	<0.001^**^	*r *= 0.88
**P Lens_D_max_**	Gy	9.9 ± 2.7	8.3 ± 2.9	1.6(1.31→1.89)	11.078	<0.001^**^	*d *= 1.75
**P Lens_D_0.03_**	Gy	7.5 ± 2.1	5.8 ± 2	1.7(1.47→1.92)	14.963	<0.001^**^	*d *= 2.37
**Lacrimal_D_mean_**	Gy	8.4 ± 1.9	7.5 ± 2	0.9(0.76→1.05)	12.29	<0.001^**^	*d *= 1.94
**Lacrimal_V_6Gy_**	%	55.6 ± 13.7	37.2 ± 12	18.4(16.8→19.87)	24.159	<0.001^**^	*d *= 3.82
**Lacrimal_V_10Gy_**	%	32.3 ± 12	23.7 ± 10.3	8.6(7.46→9.79)	14.94	<0.001^**^	*d *= 2.36
**Lacrimal_V_15Gy_**	%	11.4 ± 8.6	14.3 ± 8.4	−2.9(−3.51→−2.18)	−8.655	<0.001^**^	*d *= −1.37
**Plan Quality**							
**N(field)**		3[2,3]	4[3,4]	−1(−1.19→−0.06)	−5.203	<0.001^**^	*r *= −0.95
**Dr**	Gy/mm	2[1.9,2.2]	2.3[2,2.5]	−0.3(−0.47→−0.03)	−5.266	<0.001^**^	*r *= −0.88
**Pb_Eye**	mm	8[8,8.5]	5.5[5.5,6]	2.5(1.84→3.16)	5.559	<0.001^**^	*r *= 0.88
**HI**		0.076 ± 0.006	0.08 ± 0.006	−0.004(−0.005→−0.002)	−4.602	<0.001^**^	*d *= −0.73
**V_5Gy‐PTV_**	cc	1237.7 ± 162.5	1195.5 ± 156.4	42.2(39.55→44.93)	31.731	0.001^**^	*d *= 5.02
**V_2Gy‐PTV_**	cc	1691.1 ± 217.7	1566.3 ± 203.98	124.8(118.61→131)	40.739	0.001^**^	*d = *6.44

***Notes***: Data: Mean ± SD (normal) or Median [IQR] (non‐normal).

Full parameters: Supplementary Tables S4.

** *P *< 0.00156 (Bonferroni‐corrected).

## 2 METHODS

### 2.1 Patient characteristics, simulation, and contouring

This retrospective analysis enrolled 40 consecutive patients with brain metastases who received WBRT between January 2024 and May 2025. The cohort comprised 32 males and eight females, with a median age of 63 years (range: 47–85). Exclusion criteria were extracranial disease extension or prior cranial irradiation. All patients were immobilized in the supine position using a thermoplastic mask with a K82‐type integrated fixation system (Ruikang'an Medical Technology, China). Simulation CT scans were acquired on an XHCT‐16 scanner (Shandong Xinhua Medical Equipment Co., Ltd.) in helical mode with a 2.5 mm slice thickness. The clinical target volume (CTV) included the entire brain parenchyma, meninges, and any macroscopic tumors. The planning target volume (PTV) was generated by applying a 3 mm isotropic margin to the CTV. Organs at risk (OARs) were contoured per institutional protocols, encompassing lenses, lens planning risk volumes (PRV_lens; 3 mm isotropic expansion), lacrimal glands, eyes, optic nerves, optic chiasm, parotid glands, brainstem, spinal cord, and skin (defined as a 3 mm subcutaneous rim). All contours were independently verified by a senior radiation oncologist and a second clinician. Detailed patient characteristics are summarized in Supporting Materials Table S1.

### 2.2 Plan design and experimental cohorts

**FIGURE 1:**
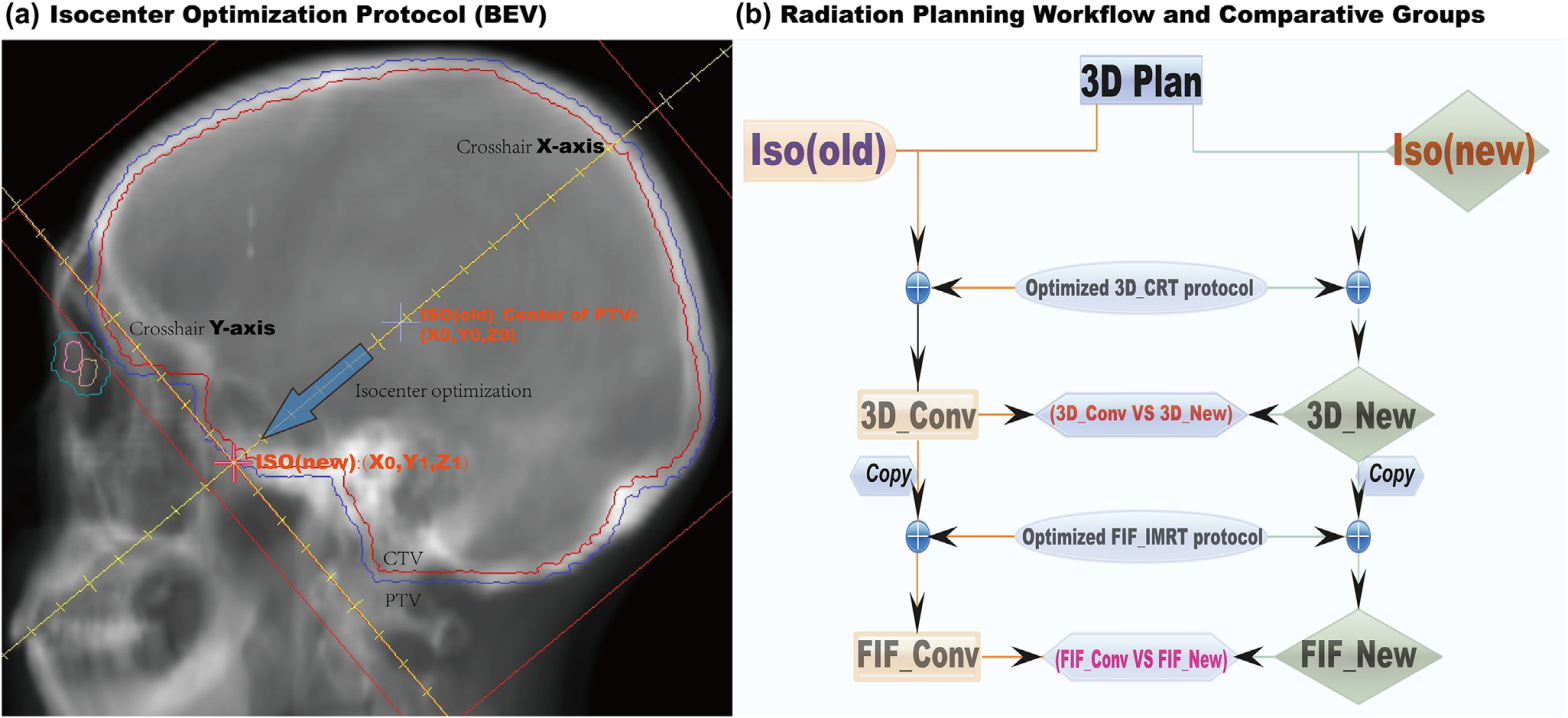
Isocenter and treatment planning optimization.

All plans were generated in the MONACO TPS (v5.11, Elekta AB, Sweden) for an Elekta Precise linac (6 MV, MLC leaf width: 10 mm at isocenter), with output calibrated to 1 MU = 1 cGy at Dmax under reference conditions. The isocenter optimization strategy is illustrated in Figure 1A. For each patient, the original isocenter (ISO_old) was placed at the geometric center of the PTV. In the beam's eye view (BEV) of the lateral fields, the isocenter was shifted anteriorly along the collimator's longitudinal axis until it intersected the anterior edge of the PTV, defining the new isocenter (ISO_new). The mean displacement vector magnitude was 56.8 mm, with components of −34 mm (superior‐inferior) and +45.6 mm (anterior‐posterior) in the patient coordinate system (X: left‐right, Y: superior‐inferior, Z: anterior‐posterior).

Four WBRT plans were generated per patient using Monte Carlo dose calculation (Grid spacing: 2 mm, statistical uncertainty: 1%). The planning target volume (PTV) prescription was 30 Gy delivered in 10 fractions. Plans were categorized into two cohorts (Figure 1B):

#### 2.2.1 Cohort 1: 3D‐CRT technique

Conventional plan (3D‐Conv) versus Isocenter‐optimized plan (3D‐New):

Both 3D‐CRT plans utilized opposed fields (90 ± 5° and 270 ± 5°) with equal weight. The 3D‐Conv plan employed ISO‐old as isocenter, while 3D‐New used ISO‐new. Collimator angles were optimized to achieve MLC shaping conforming to PTV +10 mm margin. Primary collimator jaws were set to PTV +0–5 mm with lens shielding. All 3D‐CRT plans were normalized to meet the criteria that 97% of the PTV received 30 Gy (V30Gy = 97%).

#### 2.2.2 Cohort 2: 3D‐FIF technique

Conventional plan (FIF‐Conv) versus Isocenter‐optimized plan (FIF‐New):

The FIF‐Conv plan was derived from 3D‐Conv, and FIF‐New from 3D‐New. Original fields were copied as supplementary fields (maximum two fields per lateral direction). Supplementary field MLCs were shaped to PTV +5 mm, blocking volumes receiving ≥32.4 Gy. An additional boost field (180° ± 5°) was introduced to cover underdosed regions within the PTV. This boost field was shaped to conform to the underdosed volume (with a 3 mm margin) while blocking the PRV_lens, and its monitor units were iteratively adjusted to ensure PTV coverage. Plan acceptance criteria mandated V30Gy ≥ 96% and V28.5 Gy ≥ 98% for PTV.

### 2.3 Dose and plan quality evaluation

Standard dose‐volume metrics for the planning target volume (PTV) and organs at risk (OARs) were evaluated, including coverage, homogeneity, maximum and mean doses, and volume receiving specific dose thresholds. General plan quality was assessed via monitor units and low‐dose spillage volumes.

**FIGURE 2:**
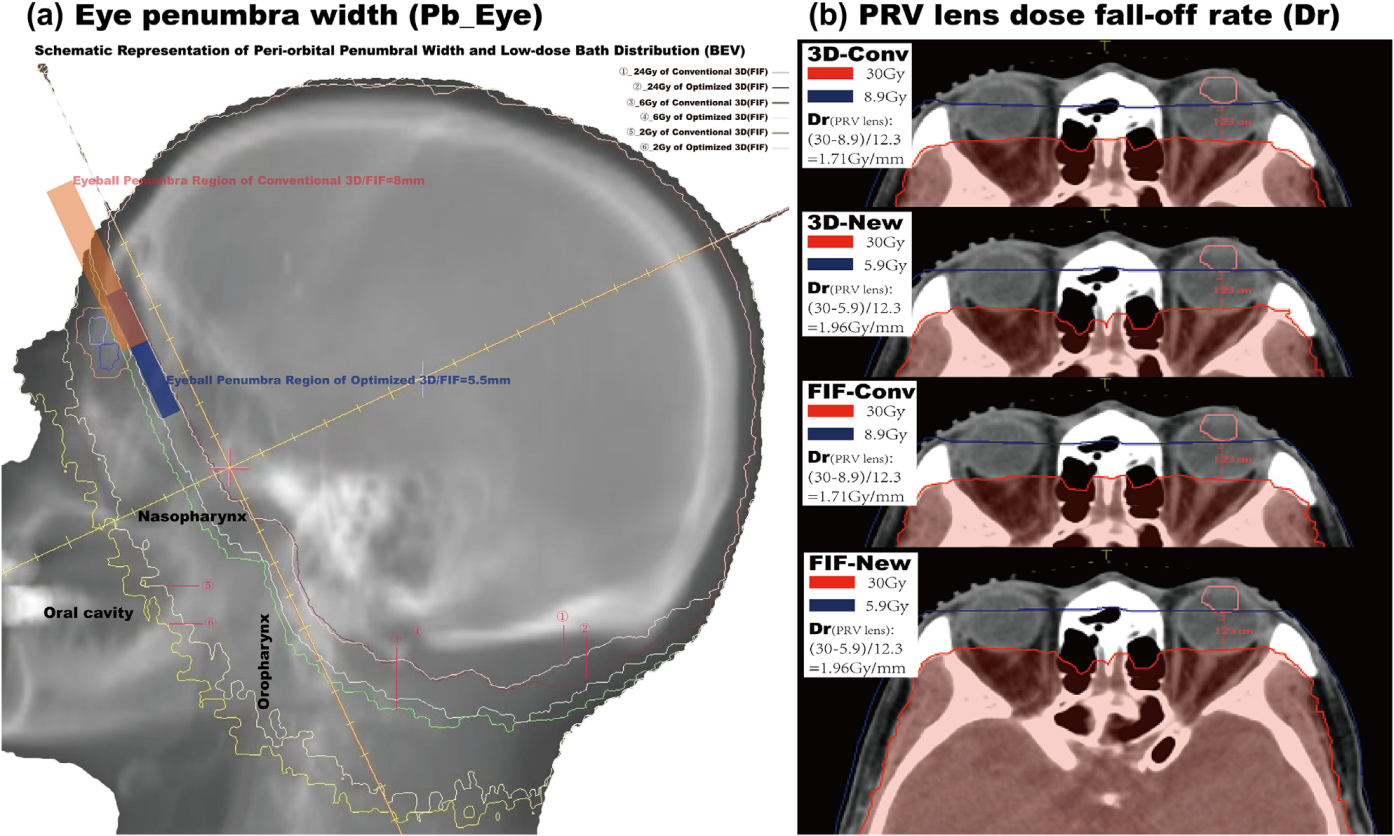
Quantification of penumbra width and dose gradient.

Critically, to directly quantify the dosimetric effect of anterior isocenter shift, two specific indices were introduced (Figure 2):

Eye Region Penumbra Width (Pb_Eye): The distance between the 80% (24 Gy) and 20% (6 Gy) isodose lines in the beam's eye view. This metric directly measures the sharpness of the anterior penumbra, which the optimization aims to reduce.

Lens Dose Fall‐off Rate (Dr): The gradient calculated from the prescription dose (30 Gy) to the maximum dose point in the PRV_lens, normalized by the perpendicular distance between them. This metric quantifies the steepness of the dose gradient protecting the lens.

Complete definitions for all standard metrics and additional indices are provided in Supplementary Materials Appendix.

### 2.4 Blinded plan quality assessment

To minimize evaluation bias, a blinded plan quality assessment was performed by three senior radiation oncologists. All plans were anonymized, randomized, and reviewed independently based solely on dose distributions and dose‐volume histograms (DVHs), with reviewers blinded to the planning technique. Clinical acceptability was strictly defined by meeting primary PTV objectives: V30Gy ≥ 96% and V28.5 Gy ≥ 98%. Plans not fulfilling these criteria were classified as having a major deviation. Secondary deviations from OAR constraints (e.g., lens Dmax < 5 Gy) were documented for qualitative analysis. Deliverability was assessed via collision avoidance checks and complexity metrics.

### 2.5 NTCP modeling, statistical analysis, and quality assurance

#### 2.5.1 NTCP modeling

Normal tissue complication probability (NTCP) modeling was performed to estimate the 5‐year risk of radiation‐induced lens opacity^36^ and the risk of acute dry eye syndrome,^21^ utilizing published dose‐response relationships. The physical mean doses (Dmean) to the lenses and lacrimal glands were converted to the equivalent dose in 2‐Gy fractions (EQD_2_) using the linear‐quadratic (LQ) model to account for differences in fractionation schemes between the source data and the present study. The conversion formula was:

$$EQD_{2}=D\;\times \;\left[\frac{\left(d+\alpha /\beta \right)}{\left(2+\alpha /\beta \right)}\right]$$

For the lens, a sensitivity analysis was conducted using (*α/β *= 0.5, 1, 1.5, 2 and 2.5 Gy). The resulting EQD_2_ values were entered into the following logistic dose‐response model^36^:

$$\;P_{\left(cataract,\;5-year\right)}=1/\left[1+exp\left(2.18045-0.1664\times EQD_{2}\right)\right]$$

For the lacrimal gland, an *α/β* value of 3 Gy was used. The Lyman‐Kutcher‐Burman (LKB) model was then applied using the following formulation^22^:

$$NTCP=\frac{1}{\sqrt{2\pi }}\;\times \int_{-\infty }^{t}e^{\frac{-x^{2}}{2}}dx$$

$$t=\frac{\left(D_{mean}-D_{50}\right)}{\left(m\times D_{50}\right)}$$

The model parameters were D_50_ = 61.2 Gy and m = 0.77.

#### 2.5.2 Statistical analysis

All statistical analyses were performed using SPSS Statistics 25.0 (IBM Corp., Armonk, NY). Paired comparisons were conducted between 3D‐Conv and 3D‐New plans, and between FIF‐Conv and FIF‐New plans. Data normality was evaluated using the Shapiro‐Wilk test. Normally distributed variables were compared with paired Student's *t*‐tests, while non‐normally distributed variables were analyzed with Wilcoxon signed‐rank tests. To control for multiple comparisons across 30 parameters, the significance level was adjusted to *α* = 0.00166 using the Bonferroni correction. Effect sizes were reported as Cohen's *d* (for parametric tests) or Wilcoxon *r* (for non‐parametric tests). Relative changes were calculated as Δ(%)=[(Conv−New)/Conv]×100%. Differences in secondary deviation rates were assessed with the McNemar test. The complete dataset of 40 patients was analyzed without outlier exclusion.

#### 2.5.3 Study quality assurance

Methodological and reporting quality was evaluated using the RAdiotherapy treatment plannINg study Guidelines (RATING) checklist,^37^ with a total score of 98% (213/216 points). The completed RATING checklist is available in Supporting Materials Table S2.

## 3 RESULTS

**FIGURE 3:**
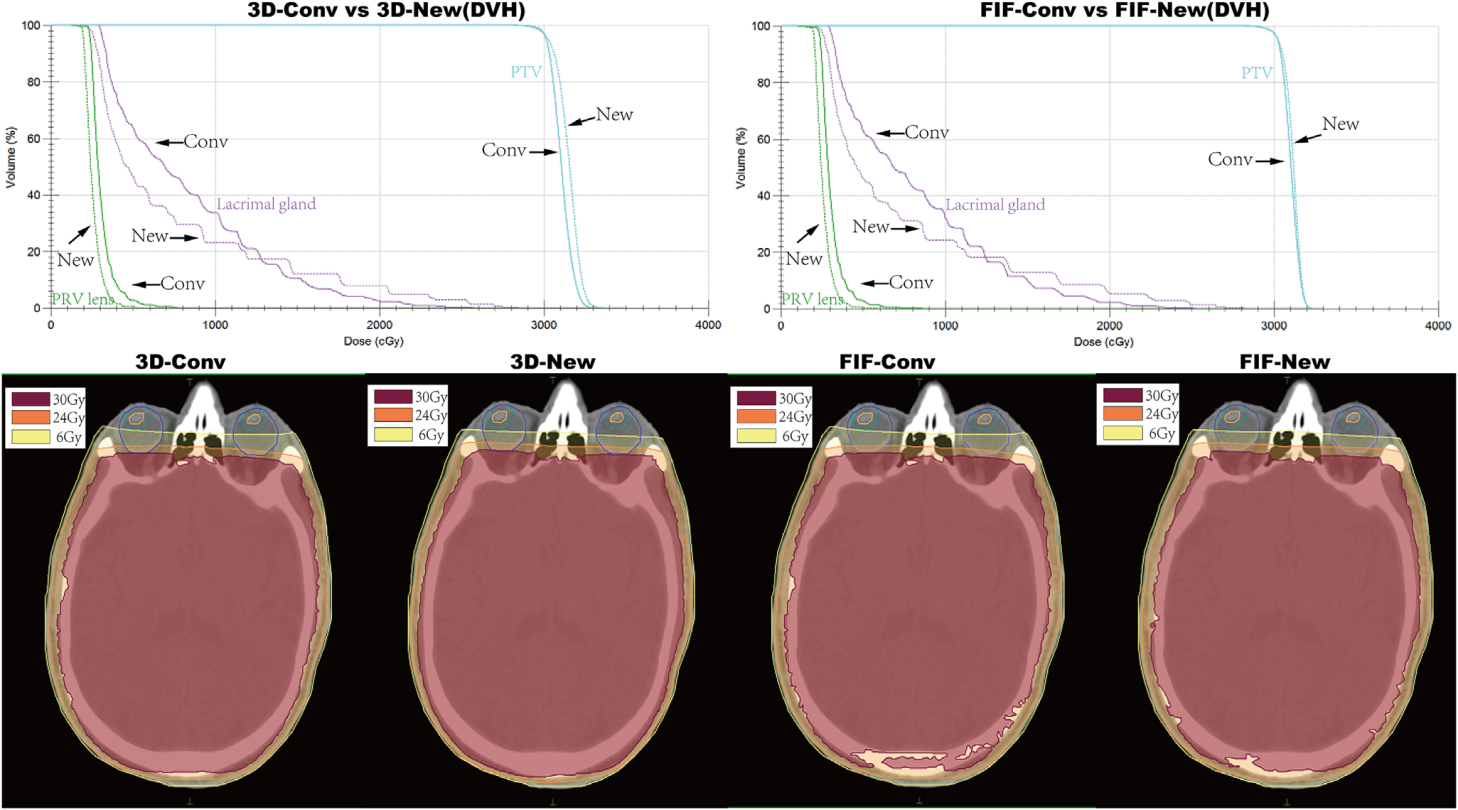
Comparative DVH and dose distribution maps.

The dose distribution outcomes are summarized in Figure 3. Isocenter optimization significantly altered dose distributions for the planning target volume (PTV), organs at risk (OARs), and overall plan quality in both the 3D‐CRT and field‐in‐field (FIF) cohorts (Supporting Materials Tables S3 and S4).

**FIGURE 4:**
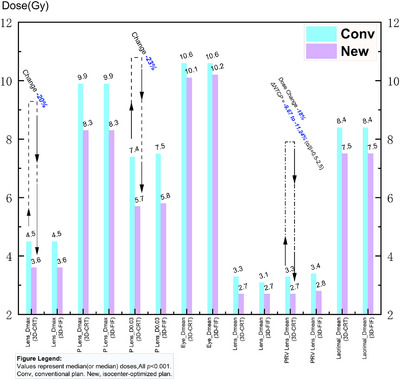
Dosimetric parameters of ocular structures.

### 3.1 Planning target volume (PTV) dosimetry

The impact of isocenter optimization on PTV dosimetry was technique‐dependent. In 3D‐CRT plans, optimization compromised dose homogeneity, as evidenced by increases in the median V33Gy (by 7 percentage points), D2% (by 0.7 Gy), and Dmax (by 0.6 Gy) (all *p* < 0.001). In contrast, FIF plans maintained consistent target coverage and homogeneity, with no significant changes in V30Gy, V33Gy, D2%, or Dmax (all *p* > 0.0017 after Bonferroni correction).

### 3.2 Organs at risk (OARs) sparing (Figure 4)

Isocenter optimization significantly reduced lens doses in both cohorts. The median lens Dmax decreased from 4.5 Gy to 3.6 Gy (a 20% reduction, *p* < 0.001), and the median PRV_lens D0.03 cm3 decreased from 7.4–7.5 Gy to 5.7–5.8 Gy (a 23% reduction, *p* < 0.001).

For the lacrimal glands, Dmean, V10Gy and V6Gy were reduced by 0.9 Gy, 8.9 percentage points, and 18.4 percentage points, respectively, whereas V15Gy increased by 2.9 percentage points (all *p* < 0.001).

### 3.3 Plan quality and delivery metrics

Optimization improved several plan quality indices. The eye region penumbra width (Pb_Eye) narrowed by 31.25%, and the lens dose fall‐off rate (Dr) increased by 15% (both *p* < 0.001). Low‐dose irradiation volumes outside the PTV (V5Gy‐PTV, V2Gy‐PTV) decreased by 3.4% and 7.4%, respectively (*p* < 0.001). However, the homogeneity index (HI) increased in 3D‐CRT plans (0.094–0.117) and, to a lesser extent, in FIF plans (0.076–0.080) (*p* < 0.001). The FIF technique required an increase in the median number of segments (from 3.0 to 4.0, *p* < 0.001).

### 3.4 Clinical risk stratification by dose thresholds

**FIGURE 5:**
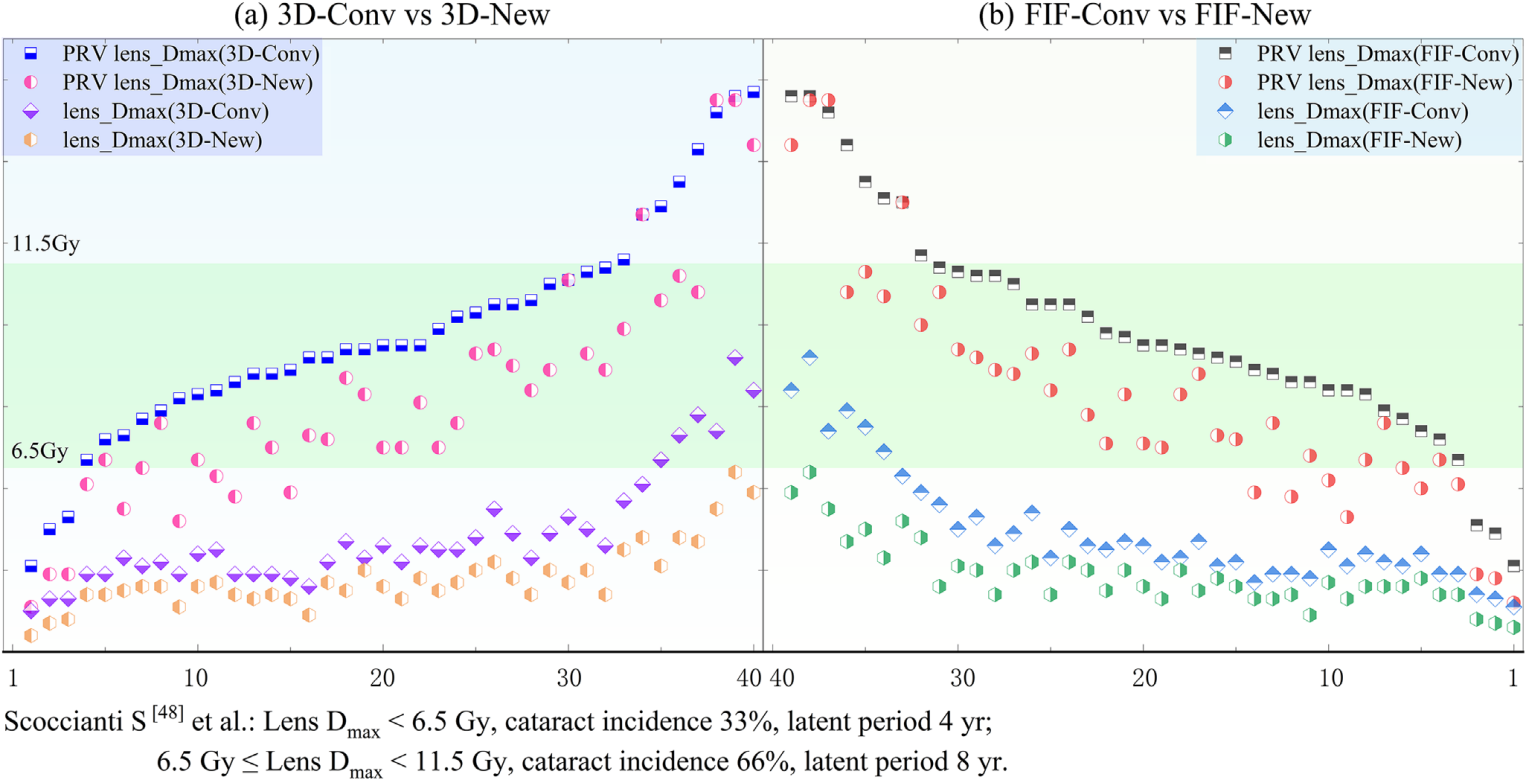
Distribution of lens dose ranges (N = 40).

Isocenter optimization substantially reduced the proportion of patients exceeding clinically relevant dose thresholds for cataract risk (Figure 5). The number of patients with a PRV_lens Dmax > 6.5 Gy decreased from 37 to 30. More notably, the number with a PRV_lens D0.03 cm^3^ > 5 Gy was reduced by 43% (from 37 to 21) in the 3D‐CRT cohort and by 38% (from 37 to 23) in the FIF cohort. Furthermore, all optimized plans maintained a lens Dmax below 6.5 Gy, and the number of patients exceeding a lens Dmax of 5 Gy was significantly reduced.

### 3.5 Plan quality compliance and deviations

All 160 generated plans (100%) satisfied the clinical acceptability criteria without major deviations. However, isocenter‐optimized plans exhibited a significantly lower frequency and severity of minor deviations relative to conventional plans (*p* < 0.001; see Supporting Materials Table S5 for details).

### 3.6 Normal tissue complication probability (NTCP) modeling

Following isocenter optimization, the projected 5‐year cataract risk was reduced by 5.98% to 11.61%, with sensitivity analysis confirming greater benefit at lower *α/β* values. This was accompanied by a relative reduction in dry eye risk of 3.6% (from 13.9% to 13.4%). (Supplementary Tables S6 and S7)

## 4 DISCUSSION

This study demonstrates that strategic anterior isocenter placement, a fundamental geometric optimization, can be quantifiably integrated into modern 3D‐CRT/FIF planning, effectively sharpening the anterior penumbra. This translation results in a hardware‐free modification that leverages the existing capabilities of any radiotherapy department.

**FIGURE 6:**
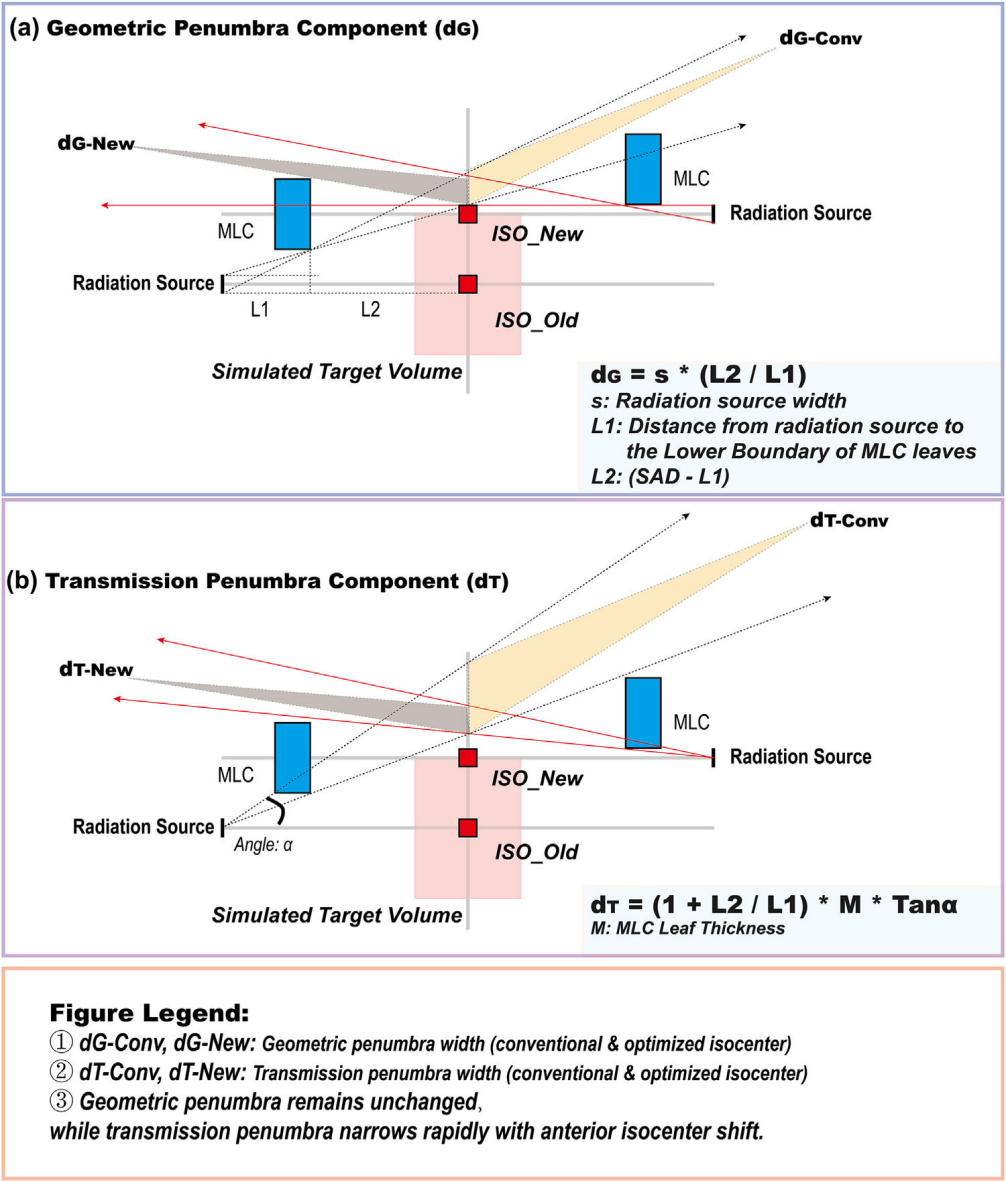
Penumbra sharpening via isocenter optimization.

The anterior isocenter shift significantly sharpened the lateral penumbra in the ocular region, resulting in a 31.25% reduction in penumbra width and a corresponding 15% increase in the dose fall‐off rate. Mechanistically, this technique compresses the penumbra by minimizing the “transmission penumbra” (Figure 6), which arises from the oblique penetration of radiation past the curved ends of the MLC leaves. As the beam transitions from initial tangency with the leaf edge to full penetration, a dose gradient is projected at the isocenter plane. Anterior isocenter positioning reduces the beam's angle of incidence relative to the leaf ends, thereby narrowing this transmission penumbra.

The efficacy of this optimization stems from a fundamental technical homology between conventional 2D and modern 3D‐conformal radiotherapy. Despite the leap from manual planning to digital reconstruction, both modalities share an identical core geometric configuration: primary reliance on a pair of opposed lateral fields to irradiate the whole brain. This continuity in beam geometry ensures that the fundamental physical mechanism—“anterior shift of the beam central axis to sharpen the anterior transmission penumbra”—remains valid and applicable. In the 2D era, this was achieved by physically moving the field crosshair^34^, ^35^; in the 3D paradigm, it is precisely replicated by shifting the isocenter anteriorly within the treatment planning system. Thus, this work transcends retrospection by identifying and leveraging the stable physical core preserved through technological evolution, to establish a universal, quantifiable rule that can be readily applied within modern digital radiotherapy platforms to improve ocularg.

Our analysis further establishes the physical optimality boundary of this approach (Figure 6B). While dosimetric benefit increases monotonically with anterior shift, the need to maintain planning target volume (PTV) coverage imposes a fundamental constraint. The optimal position is achieved when the isocenter reaches the anterior PTV edge—beyond this limit, MLC retraction would be required, leading to penumbral widening and loss of ocular sparing. Thus, the anterior PTV edge represents the quantified physical optimum. This finding provides a mechanistic basis for the historical observation that placing the beam's central axis at the lateral orbital rim improved lens sparing,^35^ generalizing the principle into a universally applicable rule for modern MLC‐based WBRT.

Consequently, the observed dosimetric improvements translated into meaningful clinical benefit: the projected 5‐year cataract risk was reduced by up to 11.61%, alongside a decrease in dry eye syndrome risk, underscoring the strategy's potential to improve quality of life for long‐term survivors.

Our findings should be contextualized within the historical evolution of WBRT planning, which reveals a selective adoption of past principles. As outlined in the Introduction, the 2D era established two seminal strategies. While the transition to 3D‐CRT seamlessly incorporated the physical penumbra compression principle (evolving into modern MLC/jaw blocking,^38^, ^39^, ^40^ the geometric optimization principle was effectively left behind. This study specifically addresses this oversight. We demonstrate that the dosimetric potential of the established “MLC+ jaw” baseline is not fully exhausted; synergistic integration with the revived geometric optimization principle yields substantial incremental benefit. A notable trade‐off emerged in conventional 3D‐CRT, where optimization increased posterior scatter, compromising dose homogeneity—a finding of clinical relevance given established links between high brain dose and neurocognitive deficits.^41^, ^42^, ^43^ Crucially, the 3D‐FIF technique successfully mitigated this inhomogeneity, preserving plan quality with only a minimal increase in delivery complexity (a single additional segment).

**FIGURE 7:**
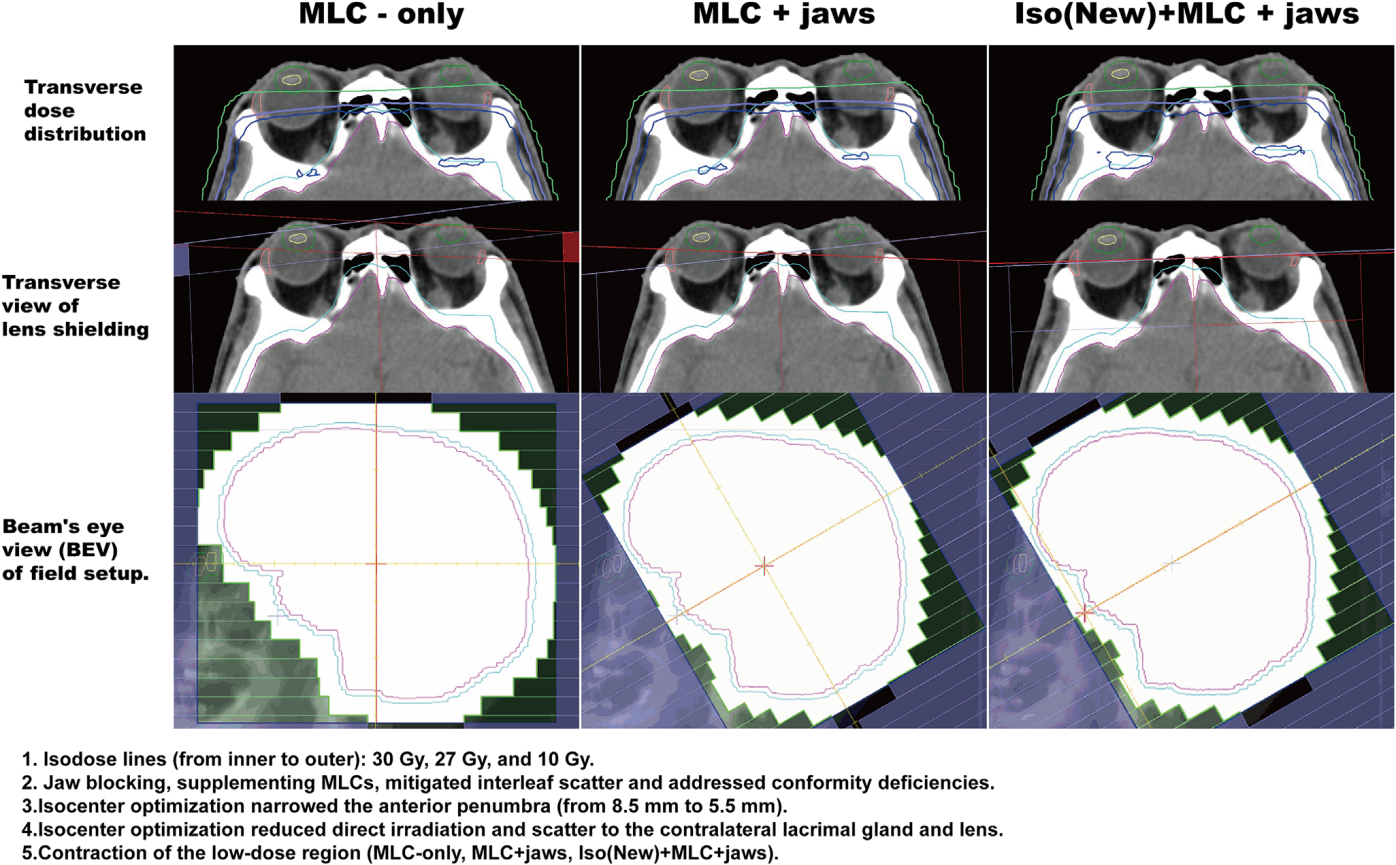
Evolution of ocular‐sparing techniques in (3D)‐WBRT.

Consequently, this study establishes and validates a “Dual‐Principle” optimization paradigm for WBRT: the foundational physical penumbra control (MLC/jaw) is synergistically augmented by the revived geometric optimization (isocenter shift). This paradigm not only demonstrates cumulative dosimetric benefit but also highlights the value of re‐examining historical principles in the context of modern radiotherapy platforms. Quantitatively, lens Dmax exhibited a stepwise reduction: 6.9 Gy (MLC‐only^40^) → 4.5 Gy (our MLC+ Jaws control) → 3.6 Gy (with Isocenter Optimization). A corresponding evolution was observed for the lacrimal gland, with its mean dose decreasing from a historical baseline of 25–26 Gy^21^ to 7.5 Gy with our integrated approach (Figure 7). This “single modification, dual benefit” was quantitatively affirmed by Normal Tissue Complication Probability (NTCP) modeling, which projected definitive risk reductions for both complications. The robustness of the cataract risk reduction was further confirmed by its stability across a wide range of *α/β* values in sensitivity analyses.

The clinical impact is further strengthened by the significant reduction in patients exceeding critical dose thresholds for the lens (Figure 5), enhancing patient risk stratification. Emerging evidence suggests that lacrimal gland toxicity may be more strongly associated with intermediate‐dose volumes (e.g., V6Gy‐V15Gy) than with mean dose.^22^ In this context, the substantial reductions we achieved in V6Gy and V10Gy are particularly significant. Furthermore, considering the parallel architecture of the lacrimal gland and the minimal absolute volume implicated, the observed modest increase in V15Gy is unlikely to compromise the net clinical benefit.

Importantly, this enhanced ocular sparing was achieved without compromising planning target volume (PTV) coverage. Our robust planning protocol, incorporating a 3 mm PTV margin and the concurrent evaluation of both lenses and their planning organ at risk volumes (PRV_lens), adequately accounted for treatment‐related uncertainties.^44^, ^45^, ^46^ This rigor resulted in a marked decrease in the proportion of plans exceeding critical dose thresholds (e.g., PRV_ lens Dmax > 6.5 Gy; PRV_ lens D0.03 cm^3^ > 5 Gy) (Figure 5). This finding aligns with established dose‐response relationships^26^, ^36^, ^47^, ^48^ and the optimization principles outlined by the ICRP,^29^ indicating a more favorable clinical risk profile. Additionally, the observed reduction in out‐of‐field low‐dose volumes (V5Gy, V2Gy) adheres to the As Low as Reasonably Achievable (ALARA) principle, potentially mitigating the risks of secondary malignancies and immune dysfunction in long‐term survivors.^49^, ^50^, ^51^, ^52^

Several limitations of this study should be acknowledged. While conducted at a single institution, the optimization is based on a fundamental geometric and physical principle (sharpening the transmission penumbra). Therefore, the dosimetric benefits and the proposed protocol are expected to be reproducible across different institutions and treatment planning systems, facilitating widespread clinical adoption. Second, the opposed‐field geometry utilized is not compatible with hippocampal avoidance. Given the critical importance of mitigating neurocognitive toxicity,^53^, ^54^, ^55^, ^56^ a compelling future direction involves integrating our isocenter optimization strategy into hippocampal‐avoidance WBRT (HA‐WBRT) platforms. This integration is particularly warranted, as lens doses reported for some contemporary HA‐WBRT techniques (5.5–6.3 Gy)^57^ exceed those achieved with our combined approach. Notably, HA‐WBRT typically requires highly modulated techniques like IMRT or VMAT. Investigating the integration and quantifying the incremental value of our geometric principle within such advanced planning systems is a necessary next step. Furthermore, the core principle—sharpening the penumbra at a target periphery—may extend to other “eccentric” clinical scenarios where sparing of the contralateral healthy organ is paramount, such as in radiotherapy for unilateral parotid carcinoma or chest wall tumors. Finally, while the employed NTCP models provide valuable quantitative risk estimates, they possess inherent limitations.^58^ A key uncertainty lies in the α/β ratios for ocular structures. Our sensitivity analysis addressed this for the lens. Reassuringly, supporting evidence suggests that the impact of such parameter uncertainty may be more limited for the lacrimal gland; in brachytherapy, the BED(10) for the lacrimal gland varied by only 1.4% over the reported α/β range, compared to a 41% variation for the lens.^59^ This indicates that comparative conclusions regarding the lacrimal gland are robust despite the acknowledged parameter uncertainty. Consequently, validation through prospective clinical trials with long‐term ophthalmologic follow‐up is essential to confirm the translation of these dosimetric improvements into tangible patient benefits.

## 5 CONCLUSION

In conclusion, anterior isocenter optimization is a practical and hardware‐free technique for WBRT. By sharpening the anterior penumbra, it achieves concurrent and significant sparing of the lens and lacrimal gland. Its straightforward implementation—a simple planning‐stage adjustment—synergizes with the FIF technique to achieve optimal ocular protection without compromising target quality. This makes it a readily implementable, high‐benefit clinical option for reducing long‐term radiotoxicities.

## AUTHOR CONTRIBUTIONS

Chen Xie, Xuemei Xia, and Haowen Pang contributed equally to this work. All authors contributed to the study conception and design. Conceptualization, Writing –original draft, Investigation, Methodology: Chen Xie; Data curation, Software, Visualization: Xuemei Xia; Formal analysis, Writing –review & editing: Haowen Pang; Funding acquisition, Project administration: Yan Zhang; Resources, Supervision: Lin Li; Validation: Simin Lu. All authors read and approved the final manuscript.

## CONFLICTS OF INTERESTS STATEMENT

The authors have no relevant financial or non‐financial interests to disclose. All authors certify that they have no affiliations with or involvement in any organization or entity with any financial interest or non‐financial interest in the subject matter or materials discussed in this manuscript.

## FUNDING INFORMATION

The authors did not receive support from any organization for the submitted work. No funds, grants, or other support was received

## ETHICS STATEMENT

This study was performed in line with the principles of the Declaration of Helsinki. Approval was granted by the Institutional Review Board of Luzhou People's Hospital (Reference Number: LLW202501009). Due to the retrospective nature of the study, which involved the analysis of pre‐existing anonymized patient data, the requirement for informed consent was waived by the ethics committee.

## Supporting information



Supporting Information

Supporting Information

Supporting Information

Supporting Information

Supporting Information

## Data Availability

The datasets generated and analyzed during the current study are not publicly available due to institutional data privacy policies but are available from the corresponding author on reasonable request.
