# Supplementary material for: Isocenter Optimization in WBRT: Concurrent Sparing of Lens and Lacrimal Gland via Anterior Penumbra Sharpening
Source: J Appl Clin Med Phys. 2026 Mar 18;27(3):e70535. doi: 10.1002/acm2.70535 (PMC13093415; doi:10.1002/acm2.70535)
Supplement: Supplementary file 2 — Supporting Information [file ACM2-27-e70535-s002.doc]

**Supporting Materials Table S2. The RATING (RAdiotherapy Treatment plannINg Study Guidelines) scoring criteria assessment for the present study.**

|  | **RATING score sheet** | **Points** | **Applicable/ relevant** | **Answer** |
| --- | --- | --- | --- | --- |
|  | **Questions for the introduction** |  |  |  |
|  | *The study aim formulated by research questions* |  |  |  |
| 1 | Does the study have a concise and precise study aim, defined with a restricted number of interconnected questions? | 10 |  | Y |
|  | *The motivation for the research questions* |  |  |  |
| 2 | Has relevant up to date literature been included to support the need for the current study? | 5 |  | Y |
| 3 | Does the study address an existing knowledge gap? | 10 |  | Y |
|  | **Questions for Materials and Method** |  |  |  |
| 4 | Is the global study design adequate for answering the posed research questions? | 10 |  | Y |
| 5 | Is the global study design described in sufficient detail for others to interpret and reproduce the results? | 5 |  | Y |
|  | *Patient cohort* |  |  |  |
| 6 | Are the inclusion and exclusion criteria of the patient cohort described? | 1 | Mandatory | Y |
| 7 | Is the clinical patient information of the cohort presented, including disease type, site(s) and clinical staging? | 1 | Mandatory | Y |
| 8 | Is the included number of patients stated, explained and justified? | 1 | Mandatory | Y |
| 9 | Has there been consideration of the need for ethical and/or legal approval for the study and if needed, is there a statement about this? | 5 |  | Y |
|  | *Imaging Procedures* |  |  |  |
| 10 | Have the scanning parameters been reported in sufficient detail (image modalities, equipment model, slice thickness, voxel size, patient position (e.g. head first, supine, etc.)? | 1 |  | Y |
| 11 | Has the applied immobilisation equipment been described, (e.g. vendor and type, standard settings, etc.)? | 1 |  | Y |
|  | *Treatment Machine and Settings* |  |  |  |
| 12 | Have the treatment machine and relevant parameters been described with sufficient detail (model, beam energy, MLC, etc.)? | 1 |  | Y |
| 13 | Have the MU reference conditions been defined? | 1 |  | Y |
|  | *Definition of Targets and OARs* |  |  |  |
| 14 | Has GTV definition been described in sufficient detail, with references if possible? | 1 |  | N |
| 15 | Has CTV definition been described in sufficient detail, with references if possible? | 1 |  | Y |
| 16 | Has the establishment of PTVs (or alternatively robustness settings) been described in sufficient detail? | 1 |  | Y |
| 17 | Have PTV sizes in the patient cohort been described? | 1 | Supplementary Table S5. Baseline characteristics of the patients | Y |
| 18 | Have OAR definitions been described in sufficient detail, with references if possible? | 1 |  | Y |
| 19 | Have PRV margins been described in sufficient detail, with references if available? | 1 |  | Y |
|  | *Treatment Planning System and Dose Calculation* |  |  |  |
| 20 | Have all applied dose calculation algorithms been described in sufficient detail? | 1 |  | Y |
| 21 | For any commercial software used, have the manufacturer, algorithms and specific versions been stated? | 1 |  | Y |
| 22 | Have all relevant user parameters and settings in the TPS been reported, e.g. beams, dose grid, control point spacing? | 1 |  | Y |
| 23 | Have all volumes been evaluated with the same software/methodology? | 1 |  | Y |
|  | *Planning Aims and Optimisation* |  |  |  |
| 24 | Are clear planning aims defined, including imposed hard constraints and planning objectives (with or without soft constraints)? | 5 | Mandatory | Y |
| 25 | Has the ranking of planning objectives (priorities) been described? (5 points, mandatory) | 5 | Mandatory | Y |
| 26 | Is the dose prescription clearly defined? | 10 | Mandatory | Y |
| 27 | Is there a description of the applied optimisation process, including the handling of all objectives with their ranking? | 5 | Mandatory | Y |
| 28 | If manual intervention during or after optimisation is allowed, has this been described? | 1 |  | Y |
|  | *Bias Mitigation* |  |  |  |
| 29 | Have enough study details been provided such that bias issues could be noted? | 5 | Mandatory | Y |
| 30 | Has bias been sufficiently mitigated to reliably answer the posed research question? | 10 | Mandatory | Y (8Points) |
|  | *Plan Acceptability– Minor and Major Protocol Deviations* |  |  |  |
| 31 | Was the procedure for assessment of plan acceptability well described? | 1 |  | Y |
| 32 | Was the procedure for assessment of minor and major protocol deviations well described? | 1 |  | Y |
|  | *Plan (re-) normalization for plan comparisons* |  |  |  |
| 33 | Has plan (re-)normalisation been described sufficiently? | 1 |  | Y |
|  | *Dose–volume Parameters for plan evaluation and comparison* |  |  |  |
| 34 | Have sufficiently comprehensive dose–volume parameters been used for plan evaluations and comparisons? | 5 |  | Y |
|  | *Population-mean DVHs* |  |  |  |
| 35 | Has the algorithm for creating population-mean/median DVHs been reported? | 1 |  | Y |
| 36 | Have the definitions of confidence intervals been included? | 1 |  | Y |
|  | *Plan Evaluations by Clinicians* |  |  |  |
| 37 | Have clinicians scored plans to assess quality? | 1 |  | Y |
| 38 | Were plan comparisons by clinicians blinded? | 1 |  | Y |
|  | *Predicted TCP and NTCP* |  |  |  |
| 39 | Have any applied TCP models been described and referenced? | 1 | **Not applicable. This study focused on the dosimetric gain in lens protection via isocenter optimization within a palliative WBRT context. Thus, the emphasis was on reducing organ-at-risk toxicity (NTCP), not on comparing TCP from target dose differences.**  **Moreover, tumor control in WBRT involves multifactorial determinants—including systemic therapy, histology, and metastatic burden—making accurate prediction from physical dose parameters alone unfeasible.**。 | N |
| 40 | Have any applied NTCP models been described and referenced? | 1 |  | Y |
|  | *Plan Deliverability and Complexity* |  |  |  |
| 41 | Have methods used to assess plan deliverability and complexity been described in sufficient detail? | 1 |  | Y |
|  | *Composite Plan Quality Metrics* |  |  |  |
| 42 | Is there a sufficient basis (e.g. in the literature) for any selected composite plan quality metrics? | 1 |  | Y |
| 43 | Is there an adequate description of the calculation of the composite plan quality metrics? | 1 |  | Y |
|  | *Planning and Delivery Times* |  |  |  |
| 44 | Has measurement of planning times been described in sufficient detail? | 1 |  | Y |
| 45 | Has the establishment of delivery times been described in sufficient detail? | 1 |  | Y |
|  | *Statistical Analysis* |  |  |  |
| 46 | Have proper statistical methods been used and described in sufficient detail? | 5 | Mandatory | Y |
| 47 | In case of multiple testing for research questions, has this been handled appropriately? | 1 |  | Y |
|  | **Questions for Results** |  |  |  |
| 48 | Does the provided data contribute to (at least partly) answering all aspects of the research questions, e.g. plan acceptability, dosimetric quality, deliverability and planning and delivery times? | 10 | Mandatory | Y |
|  | *Dose Distribution Reporting* |  |  |  |
| 49 | Are complete summaries of the dose distributions in the patient cohort provided (low doses, high doses, OARs, PTV, patient, etc.)? | 5 | Mandatory | Y |
| 50 | Are tables and figures optimised to clearly present the results obtained? | 1 |  | Y |
| 51 | Have the answers to the research questions been illustrated for an example patient by providing dose distributions, DVHs, etc.? | 1 |  | Y |
|  | *Plan acceptability reporting – minor and major protocol deviations* |  |  |  |
| 52 | In case of treatment technique or planning technique comparisons, was plan acceptability reported separately for each technique? | 1 |  | Y |
| 53 | Has plan acceptability been reported in sufficient detail: how many plans were acceptable, how many were not and for what reasons (e.g. violation of hard constraints, violation of soft constraints, other reasons)? | 1 |  | Y |
| 54 | Was there adequate reporting of minor and major protocol deviations? | 1 |  | Y |
|  | *Deliverability and complexity reporting* |  |  |  |
| 55 | Has the deliverability of the plans been adequately reported? | 1 |  | Y |
| 56 | Have plan deliverability and complexity been investigated in sufficient detail in relation to the posed research questions? | 1 |  | Y |
|  | *Planning and delivery time reporting* |  |  |  |
| 57 | Have planning and delivery times been adequately evaluated and reported? | 1 |  |  |
|  | *Patient-specific Analyses Reporting* |  |  |  |
| 58 | Is there sufficient description of inter-patient variations in the results presented? | 1 |  | Y |
| 59 | Have outlier patients been reported and has any exclusion from population analyses been sufficiently motivated and explained? | 1 |  | Y |
|  | *Statistical Reporting* |  |  |  |
| 60 | Are the p-values reported appropriately? | 1 |  | Y |
| 61 | Are there confidence intervals for the appropriate parameters? | 1 |  | Y |
|  | *Questions for discussions* |  |  |  |
| 62 | Is there an overall interpretation of the data presented in the Results section as to how the posed research questions are answered? | 10 | Mandatory | Y |
|  | *Comparison with Literature* |  |  |  |
| 63 | Has the study been sufficiently discussed in the context of existing literature? | 5 | Mandatory | Y |
|  | *Clinical and Statistical Significance* |  |  |  |
| 64 | Does the discussion focus on statistically significant results? | 1 |  | Y |
| 65 | Is the potential clinical significance of the results clearly discussed (assuming practical application would be feasible)? | 5 | Mandatory | Y |
|  | *Clinical and statistical significance* |  |  |  |
| 66 | Is future clinical applicability sufficiently discussed? | 1 |  | Y |
|  | *Study Limitations* |  |  |  |
| 67 | Has the impact of the study limitations on the provided answers to the research questions been sufficiently discussed? | 10 | Mandatory | Y |
|  | *Future Work* |  |  |  |
| 68 | Has the potential future work arising from the study been discussed? | 1 |  | Y |
|  | **Questions for conclusions** |  |  |  |
| 69 | Do the presented conclusions represent answers to the posed research questions? | 5 | Mandatory | Y |
| 70 | Are the conclusions supported by the results? | 5 | Mandatory | Y |
| 71 | Are the conclusions a fair summary of all results? | 5 | Mandatory | Y |
|  | **Questions for Supplementary** |  |  |  |
|  | *Supplementary Materials* |  |  |  |
| 72 | Is the information presented in the supplementary material of sufficient relevance? | 1 |  | Y |
| 73 | Is the presentation of the included information of sufficient quality, including readability? | 1 |  | Y |
| 74 | Has sufficient underlying data been made available or a willingness to share data been indicated, within local data sharing restrictions? | 5 | Mandatory | Y |
|  | **RTING remarks** |  |  |  |
| 75 | Is the RATING score added to the manuscript? | 5 | Mandatory | Y |
| 76 | Is the accompanying question table added to the cover letter or the supplementary material? | 1 |  | Y |
